# Tailoring Metal–Oxide Interfaces via Selectively CeO_2_-Decorated Pd Nanocatalysts with Enhanced Catalytic Performance

**DOI:** 10.3390/nano15030197

**Published:** 2025-01-27

**Authors:** Ziwen Liu, Guizhen Zhang, Lijuan Niu, Zaicheng Sun, Zhenguo Li, Hong He

**Affiliations:** 1 Beijing Key Laboratory for Green Catalysis and Separation, College of Materials Science & Engineering, Beijing University of Technology, Beijing 100124, China; liuzw2023.chji@sinopec.com (Z.L.);; 2 Institute of Engineering Technology, Sinopec Catalyst Co., Ltd., Beijing 101111, China; 3 Carbon Energy Technology Co., Ltd., Beijing 102400, China; nlj@carbonenergy.com.cn; 4 Center of Excellence for Environmental Safety and Biological Effects, Beijing Key Laboratory for Green Catalysis and Separation, Department of Chemistry, College of Chemistry and Life Science, Beijing University of Technology, Beijing 100124, China; sunzc@bjut.edu.cn; 5 China Automotive Technology and Research Center Co., Ltd., Tianjin 300300, China

**Keywords:** metal–oxide interface, selectively coating, thermal stability, three-way catalytic activity

## Abstract

Metal–oxide interfaces play a prominent role in heterogeneous catalysis. Tailoring the metal–oxide interfaces effectively enhance the catalytic activities and thermal stability of noble metal catalysts. In this work, polyvinyl alcohol-protected reduction and L-arginine induction methods are adopted to prepare Pd catalysts (Pd/Al_2_O_3_-xCeO_2_) that are selectively decorated by CeO_2_, which form core–shell-like structures and generate more Pd-CeO_2_ interfacial sites, so that the three-way catalytic activity of Pd/Al_2_O_3_-xCeO_2_ catalysts is obviously significantly enhanced due to more adsorption oxygen at the interface of Pd-CeO_2_ and good low-temperature reducibility. At the moment, the Pd/Al_2_O_3_-xCeO_2_ catalysts exhibit excellent thermal stability after being calcined at 900 °C for 5 h, owing to the Pd species being highly redispersed on CeO_2_ and part of the Pd species being incorporated into the lattice of CeO_2_. This is a major reason for the Pd/Al_2_O_3_-xCeO_2_ catalysts to maintain high catalytic activity after aging at high temperatures. It is concluded that the metal–oxide interfaces and the interaction between Pd NPs and CeO_2_ are responsible for the excellent catalytic performance and stability of Pd/Al_2_O_3_-xCeO_2_ catalysts in three-way reactions.

## 1. Introduction

Heterogeneous catalyst systems consisting of metals and oxide supports are widely used. Particularly, nano-sized metal particles have been extensively researched in the field of catalysis in recent years due to their high surface to volume ratios and effective catalytic performance [1,2]. Particularly when dispersed on oxide supports, their catalytic activities are greatly enhanced compared to those of pure metal particles or oxide supports because of the interaction between noble metal particles and oxide [3]. It is generally believed that the catalytic active site is located at the metal–oxide interface, which suggests that the rational design of the metal–oxide interface is critical for a highly efficient catalytic system [4]. However, it is limited to the contact area of the metal–oxide interface in traditional supported catalysts.

In addition, owing to their high surface area and surface energy, noble metal nanoparticles (NPs) on supported catalysts tend to aggregate into larger particles to minimize their surface energy, especially during catalytic reaction at high temperatures, which could easily lead to their deactivation or the loss of catalytic activity [5,6]. For example, palladium (Pd) is the main active ingredient in most three-way catalysts (TWCs), usually loaded on an Al_2_O_3_ support, which can convert CO, HC, and NOx pollutants into non-toxic substances for controlling automobile exhaust emissions [7,8]. In addition, as one of the most commonly used reducible oxides, CeO_2_ is widely used because of its high oxygen storage capacity and excellent redox properties, and it also plays an important role in promoting the dispersion of noble metals in TWCs [9]. However, in order to meet increasingly stringent emission standards over the life of vehicles, the TWC is often installed in a close-coupled position near the exhaust manifold outlet to allow for quickly achieving light-off temperature after a cold start. During the warming-up of the engine, the temperature in this position can sometimes even reach up to 1000 °C [8]. Such a high temperature severely leads to catalysts’ deactivation or the sintering of noble metal [10,11].

In summary, in order to prepare catalysts with high activity and excellent thermal stability, various strategies have been reported. In particular, core–shell nanostructures with inorganic oxide coatings are the ideal solution approaches, and have been widely researched recently [12,13,14,15,16,17,18,19,20]. Joo et al. reported the design of a high-temperature-stable model catalytic system that consisted of a Pt metal core coated with a mesoporous silica shell (Pt@mSiO_2_), which showed high thermal stability against metal particle agglomeration or sintering even after being subjected to 750 °C in air. The Pt@mSiO_2_ catalysts still keep the structure of Pt cores encaged within silica shells [12]. However, the etching process requires tedious steps and is time-consuming. Li and coworkers designed the hydrothermal stability of TWCs of core–shell Pd@Ce_0.5_Zr_0.5_O_2_/Al_2_O_3_ catalysts, and the Pd NPs remained isolated when the catalysts were calcined at 1050 °C in the presence of 10% water, but the catalytic activity of a fresh Pd@Ce_0.5_Zr_0.5_O_2_/Al_2_O_3_ catalyst is much lower than Pd/Al_2_O_3_ [10]. This reduction in activity can be attributed to the blocking of active sites by the protective layer, which is a major deterrent to the practical utilization of these catalysts. Lu et al. designed a coking- and sintering-resistant palladium catalyst by atomic layer deposition (ALD), which can enhance the catalytic activity and stability of the Pd/Al_2_O_3_catalyst for the oxidative dehydrogenation of ethane. ALD, an advanced technology, can precisely control the thickness and position of the overcoating by a self-limiting growth process [21]. However, the method needs special instruments, and the precursors used are expensive, so they cannot be prepared on a large scale.

To overcome these challenges mentioned above, we report a facile way to precisely synthesize Pd/Al_2_O_3_-xCeO_2_ catalysts with excellent catalytic activity and thermal stability, in which CeO_2_ selectively decorated the Pd NPs supported on Al_2_O_3_ by the L-arginine induction method to enlarge the metal–oxide interface. The physicochemical properties of the synthesized catalysts were characterized by a series of test methods, including TEM, XRD, BET, H_2_-TPR, XPS, O_2_-TPD, CO pulse adsorption, and so on. The results show that the oxygen adsorption capacity, low-temperature reducibility, and thermal stability of the catalysts can be significantly improved by constructing a rich Pd-CeO_2_ interface. We believe that this work could provide a more fundamental understanding and means to improve catalytic performance by modulating the metal–oxide interface.

## 2. Experiment

### 2.1. Materials

Poly(vinyl pyrrolidone) (PVP, MW = 58,000), polyvinyl alcohol (PVA, 98–99% hydrolyzed, low molecular weight), aluminum oxide (Al_2_O_3_, NanoTek, 99.5%), and L-arginine (98.0%) were obtained from Alfa Aesar (Shanghai, China) Chemicals Co., Ltd. Ascorbic acid (AR 99.0%) and KBr (SP 99.0%) were purchased from Sinopharm (Beijing, China) Chemical Reagent Co., Ltd. Na_2_PdCl_4_ (99.9%) was purchased from J&K Chemical (Beijing, China). Ce(NO_3_)_3_·6H_2_O (AR 99.0%) and NaBH_4_ (AR 98.0%) were obtained from Tianjin Fuchen Chemical Reagents Factory (Tianjin, China).

### 2.2. Catalyst Preparation

We undertook the synthesis of Pd/Al_2_O_3_-xCeO_2_ fresh catalysts through polyvinyl alcohol-protected reduction and L-arginine induction methods. First, Pd/Al_2_O_3_ catalysts were synthesized by a PVA-protected reduction strategy [22]. The typical steps are as follows: The required amount of PVA (noble metal/PVA mass ratio = 1:1.5) was added to an aqueous solution of Na_2_PdCl_4_ and stirred vigorously in an ice bath for 20 min. A rapid injection of NaBH_4_ (2 g/L, molar ratio of noble metal/NaBH_4_ = 1:5) produced a dark-colored sol. A certain weight of Al_2_O_3_ power was then added to the dark-colored sol under stirring until complete adsorption. The resulting mixture was filtered, washed with deionized water, and dried at 80 °C overnight. The loading of Pd species was adjusted by controlling the additional amount of Al_2_O_3_ power. The Pd/CeO_2_ catalyst was prepared by similar methods just by replacing Al_2_O_3_ with CeO_2_.

Then, the L-arginine induction method was used to synthesize Pd/Al_2_O_3_-xCeO_2_ catalysts by CeO_2_, which selectively decorated the Pd nanoparticles supported on the Al_2_O_3_. The detailed process is as shown below: A desired amount of dried Pd/Al_2_O_3_ catalysts was dispersed in 30 mL deionized water under vigorous stirring for 1 h and followed by dropping in 10 mL L-arginine solution (L-arginine/Ce(NO_3_)_3_·6H_2_O molar ratio = 3.5:1 in 10 mL H_2_O) at room temperature under vigorous stirring for 40 min. Next, a certain molar amount of Ce(NO_3_)_3_·6H_2_O was dissolved in a mixture solution of 20 mL H_2_O and 20 mL ethanol, then was added into the above system under vigorous stirring for 10 min. Finally, the mixture was heated at 80 °C for 12 h. The obtained mixture was filtered, washed with deionized water, and dried at 120 °C overnight. Finally, the Pd/Al_2_O_3_-xCeO_2_ fresh catalyst was obtained by calcining the sample from RT to 500 °C in the air at a ramp rate of 5 °C/min and maintaining this temperature for 3 h. The content of CeO_2_ was regulated by changing the additional amount of CeO_2_.

In addition, the Pd/Al_2_O_3_-xCeO_2_-JZ catalyst was synthesized by a conventional wet impregnation method. First, a certain amount of dried Pd/Al_2_O_3_ catalysts was dispersed in certain molar amounts of Ce(NO_3_)_3_·6H_2_O solution under vigorous stirring, and then the slurry was vaporized with sonication (60 kHz) at 80 °C, followed by drying at 120 °C for 12 h.

The methodology developed by Xia’s team was employed to fabricate Pd nanoparticles (NPs) supported on Al_2_O_3_, with diameters of 12 nm and 19 nm, for use as Pd/Al_2_O_3_ catalysts (designated as x nm Pd/ Al_2_O_3_) [1]. The synthesis procedure is outlined as follows: An aqueous solution containing 50 mg of polyvinylpyrrolidone (PVP), 60 mg of ascorbic acid, and either 300 mg or 450 mg of potassium bromide (KBr) was introduced into a flask, amounting to a total volume of 8.0 mL. This solution was preheated to 80 °C in an oil bath while being subjected to magnetic stirring for a duration of 10 min. Following this, 3.1 mL of a sodium tetrachloropalladate (Na_2_PdCl_4_) aqueous solution with a concentration of 0.0625 M was introduced into the mixture. The reaction was allowed to proceed for 3 h at a constant temperature of 80 °C. Afterward, the resulting product was isolated via centrifugation and subsequently rinsed with acetone and ethanol to eliminate any surplus PVP. The purified Pd NPs were then redispersed in 20 mL of ethanol under vigorous stirring, into which a measured quantity of Al_2_O_3_ support was added until complete adsorption was achieved. The resulting mixture was centrifuged, washed with deionized water, and dried at 120 °C for 12 h to prepare the x nm Pd/ Al_2_O_3_ catalysts for further use. The x nm Pd/ Al_2_O_3_-xCeO_2_ and x nm Pd/ Al_2_O_3_-xCeO_2_-JZ catalysts were synthesized using L-arginine-induced methods and the conventional wet impregnation technique previously described.

All of the fresh catalysts were calcined at 500 °C for 3 h in a furnace. For the aged catalysts, the catalysts were calcined at 900 °C in air for 5 h to obtain aged catalysts. The actual loading of the catalysts was determined by ICP-AES as shown in Table S1.

### 2.3. Evaluation of Catalytic Performance

The three-way catalytic performances over the catalysts were evaluated in a fixed-bed reactor (i.d. = 8 mm). The catalyst (200 mg) was fixed in the middle of the reactor by packing quartz wool at both ends. The reaction temperature was monitored by a thermocouple located at the bed of the catalyst. The reaction mixture contained 1.6% CO, 0.05% HC (propane/propene = 2/1), 0.1% NO_x_, 1.0% O_2_, 0.23% H_2_, and N_2_ as balance gas. The corresponding space velocity (SV) was 300,000 mL·(g·h)^–1^. The concentrations of CO, NO_x_, and HC in the inlet and outlet of the reactor were analyzed on a Horiba MEXA-584L automobile emission analyzer. In this work, *T*_90_ (corresponding to the temperature at which the 90% conversion of a given compound is completed) was used to evaluate the activities of different catalysts.

### 2.4. Characterizations

The catalysts were characterized using a series of test methods such as TEM, XRD, BET, H_2_-TPR, XPS, O_2_-TPD, and so on. The detailed catalyst characterizations and catalytic evaluation procedures are described in the Supplementary Materials.

## 3. Results and Discussion

### 3.1. Characterization of Fresh Catalysts

#### 3.1.1. Morphology Analysis

**Figure 1:**
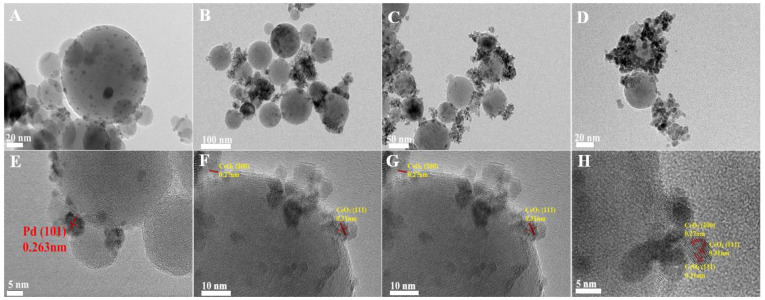
TEM and HRTEM of fresh (**A**,**E**) Pd/Al_2_O_3_, (**B**,**F**) Pd/Al_2_O_3_-1.2CeO_2_, (**C**,**G**) Pd/Al_2_O_3_-2.4CeO_2_, (**D**,**H**) Pd/Al_2_O_3_-3.0CeO_2_ catalysts.

The high-resolution transmission electron microscopy (HRTEM) images of the irregularly shaped Pd nanoparticles are depicted in Figure S1. These nanoparticles exhibited an average diameter of approximately 4 nm, with a lattice spacing of 0.22 nm, which is consistent with the (111) planes of Pd nanocrystals [1]. Following the deposition of these Pd nanoparticles onto Al_2_O_3_, the transmission electron microscopy (TEM) and HRTEM images of the pristine Pd/Al_2_O_3_ catalyst are presented in Figure 1A,E, respectively. The Pd nanoparticles were uniformly dispersed on the Al_2_O_3_ support, with a lattice spacing of 0.263 nm. This observation is indicative of the (101) crystallographic plane of PdO, suggesting that the metallic Pd nanoparticles were oxidized to PdO upon heating to 500 °C.

TEM images of the fresh Pd/Al_2_O_3_-xCeO_2_ catalysts are presented in Figure 1B–D,F–H, revealing numerous tiny CeO_2_ NPs supported on the surface of Pd/Al_2_O_3_, resembling an incomplete shell. Given that the size of the Pd nanoparticles is approximately 4 nm, which is similar to the size range of the CeO_2_ nanoparticles (3–7 nm) as determined by the Scherrer equation (Table 1), it becomes challenging to distinguish between Pd and CeO_2_ nanoparticles using TEM and HRTEM. Furthermore, the HRTEM images indicate lattice spacings of 0.27 nm and 0.31 nm, which correspond to the (200) and (111) planes of the fluorite-phase CeO_2_, respectively. To further analyze the distribution of Pd and CeO_2_ components within these catalysts, energy-dispersive X-ray spectroscopy (EDS) mapping analyses were conducted. The results for the fresh Pd/Al_2_O_3_-1.2CeO_2_ catalyst are depicted in Figure S2. It is observed that the Pd nanoparticles supported on the Al_2_O_3_ are encircled by CeO_2_ nanoparticles; however, the evidence is insufficient to conclusively demonstrate that the Pd nanoparticles on Al_2_O_3_ can be selectively decorated by CeO_2_ nanoparticles.

To conclusively demonstrate the selective decoration of Pd nanoparticles (NPs) by CeO_2_ nanoparticles (NPs), larger Pd NPs with diameters of 12 nm and 19 nm were synthesized, as shown in Figure S3. The energy-dispersive X-ray spectroscopy (EDS) mapping images of the fresh 12 nm Pd/Al_2_O_3_-1.2CeO_2_ catalysts are displayed in Figures S4–S6. These images clearly reveal the formation of a typical core–shell structure, with Pd NPs surrounded by a multitude of CeO_2_ NPs, signifying the creation of a core–shell configuration and an abundance of Pd-CeO_2_ interfaces. A similar phenomenon was observed in the 19 nm Pd/Al_2_O_3_-1.2CeO_2_ catalysts, where Pd NPs were centrally located and enveloped by a shell of dispersed CeO_2_ NPs, confirming core–shell structure formation. However, the elemental distribution in the fresh catalysts of 12 nm Pd/Al_2_O_3_-2.4CeO_2_-JZ and 19 nm Pd/Al_2_O_3_-1.2CeO_2_-JZ was also examined, as depicted in Figures S7–S9. It was evident that CeO2 was scattered across the surface of the Al_2_O_3_, following the distribution of Al_2_O_3_, which suggests that CeO_2_ could not selectively decorate Pd NPs when using the traditional wet impregnation method. Based on the aforementioned results, it can be concluded that Pd NPs supported on Al_2_O_3_ can be selectively decorated by CeO_2_ NPs through the L-arginine induction method, leading to the formation of numerous Pd-CeO_2_ interfaces.

#### 3.1.2. Pore Structure Properties

Analysis of the nitrogen adsorption–desorption isotherms revealed that all catalysts exhibited a Type IV isotherm, as shown in Figure S10. The presence of an H3 hysteresis loop without a saturation plateau at relatively high pressures (0.7–1.0) indicates the presence of irregular mesopores. The specific surface areas and total pore volumes for the Pd/Al_2_O_3_, Pd/Al_2_O_3_-xCeO_2_, and Pd/Al_2_O_3_-2.4CeO_2_-JZ catalysts are detailed in Table 1. For the fresh Pd/Al_2_O_3_-xCeO_2_ catalysts, the surface area increased while the total pore volume decreased with the gradual increase in CeO_2_ content. When compared to the Pd/Al_2_O_3_ and Pd/Al_2_O_3_-2.4CeO_2_-JZ catalysts, the Pd/Al_2_O_3_-xCeO_2_ catalysts displayed a larger surface area and total pore volume, ranging from 56 to 71 m^2^/g and from 0.248 to 0.220 cm^3^/g, respectively. It is generally accepted that the increased pore volume and specific surface area contribute significantly to the enhanced catalytic performance observed in the fresh Pd/Al_2_O_3_-xCeO_2_ catalysts. However, when considering the subsequent catalytic activity, the Pd/Al_2_O_3_-1.2CeO_2_ catalyst demonstrated the best performance, despite having a smaller surface area compared to the Pd/Al_2_O_3_-2.4CeO_2_ and Pd/Al_2_O_3_-3.0CeO_2_ catalysts. This suggests that surface area is not the primary factor influencing catalytic activity.

#### 3.1.3. Crystal Phase Analysis

**Figure 2:**
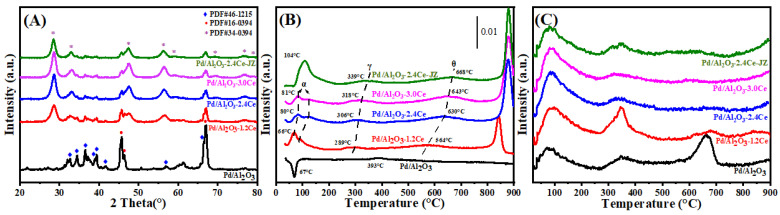
(**A**) XRD patterns, (**B**) H_2_-TPR profiles, and (**C**) O_2_-TPD profiles of the fresh catalysts.

Figure 2A presents the X-ray diffraction (XRD) patterns for both fresh and aged Pd/Al_2_O_3_, Pd/Al_2_O_3_-xCeO_2_, and Pd/Al_2_O_3_-2.4CeO_2_-JZ catalysts, with the crystallite sizes of the CeO_2_ phases tabulated in Table 1. In the fresh catalysts, the diffraction patterns attributable to Al_2_O_3_ and CeO_2_ correspond to the δ-Al_2_O_3_ phase (JCPDS No. 46-1215) and the cubic phase (JCPDS No. 34-0394), respectively. Notably, no distinct Pd phase peaks are observed in these fresh catalysts, which can be attributed to the relatively low Pd loading or the fine dispersion of Pd species on the Al_2_O_3_ surface.

#### 3.1.4. Low-Temperature Reducibility Analysis

H_2_-TPR profiles of the Pd/Al_2_O_3_, Pd/Al_2_O_3_-xCeO_2,_ and Pd/Al_2_O_3_-2.4CeO_2_-JZ fresh catalysts are displayed in Figure 2B. Furthermore, the values of H_2_ consumption are calculated using the CuO sample as a standard with a similar TPR procedure, and the results are summarized in Table 2.

In the case of the Pd/Al_2_O_3_ sample, a distinct negative peak is observed at 67 °C, corresponding to a hydrogen release of 26 μmol/gcat. This phenomenon can be ascribed to the decomposition of β-PdHx. Under conditions of low hydrogen pressure and ambient temperature within a hydrogen-rich environment, species like PdO are reduced to their metallic Pd form, which then adsorbs hydrogen to form β-PdHx species. Subsequently, a second peak emerges at a higher temperature of 393 °C, likely due to the reduction of a two-dimensional (2D) PdO surface phase. The formation of this 2D surface phase is hypothesized to occur during high-temperature oxidation processes [23]. This particular species is noted to be more resistant to reduction compared to the PdO species observed at lower temperatures [24].

For the fresh Pd/Al_2_O_3_-xCeO_2_ catalysts, two reduction peaks below 200 °C are observed, which are attributed to the reduction of surface oxygen species associated with Pd sites or Pd interacting with ceria at the Pd-CeO_2_ interface. This interface is considered the primary active site for Pd-supported catalysts [25]. In addition, the total H_2_ consumption below 200 °C follows the order Pd/Al_2_O_3_-1.2CeO_2_ (88.7 μmol/g_cat_) > Pd/Al_2_O_3_-2.4CeO_2_ (76.8 μmol/g_cat_) > Pd/Al_2_O_3_-3.0CeO_2_ (69.9 μmol/g_cat_). This indicates that Pd/Al_2_O_3_-1.2CeO_2_ has the best low-temperature reducibility among the others, achieving the lowest reduction temperature. The low-temperature reducibility of a catalyst is typically correlated with its adsorbed oxygen species [26]. Furthermore, the Pd/Al_2_O_3_-2.4CeO_2_-JZ sample shows a higher reduction temperature than Pd/Al_2_O_3_-xCeO_2_ catalysts at 104 °C, with a hydrogen consumption of 225.7 μmol/g_cat_, which is much larger than the theoretical value (94 μmol/g_cat_) required for reducing PdO to Pd^0^. This suggests the presence of a spillover phenomenon, where adsorbed hydrogen from the noble metal particles is transferred to the support due to the strong interaction between PdO and CeO_2_. This leads to the reduction of interfacial Ce^4+^ at low temperatures. These findings indicate that there are distinct interactions between Pd nanoparticles and CeO_2_ in the Pd/Al_2_O_3_-xCeO_2_ and Pd/Al_2_O_3_-2.4CeO_2_-JZ catalysts prepared by different preparation methods.

The reduction peaks at higher temperatures (γ) were observed for the catalysts, which can be attributed to the hydrogen consumption of surface CeO_2_ that is in close contact with the Pd species. Furthermore, a reduction peak (θ) at 550–700 °C is likely due to the reduction of surface Ce^4+^ ions that are distant from Pd species [27]. Above 800 °C, the reduction peaks can be associated with the bulk reduction of CeO_2_. The H_2_-TPR results indicate that the Pd-CeO_2_ interface, formed by the L-arginine induction method, exerts a more pronounced influence on the redox properties of the materials.

#### 3.1.5. Oxygen Species Analysis

It is widely accepted that the capacity of catalysts to adsorb and activate oxygen species is a critical factor in heterogeneous catalysis. O_2_-TPD measurements were conducted on the fresh catalyst, as depicted in Figure 2C. The adsorbed oxygen typically undergoes the following sequence of transformations: O_2_ (ad) → $O_{2}^{-}$(ad) → $O^{-}$(ad) →O^2−^(lattice) [28]. Generally, the desorption peak below 400 °C is attributed to the surface oxygen species (O_ad_), while those in the range of 600 to 700 °C correspond to the decomposition of palladium oxide into metallic palladium [29], which occurs at a significantly lower temperature than the bulk PdO (approximately 790 °C). This reduction in temperature is attributed to the fact that nanometer-sized materials typically have lower melting points than their bulk counterparts, owing to their increased surface area to volume ratio and elevated surface energy [30].

When comparing the O_ad_ desorption peak areas between the Pd/Al_2_O_3_, Pd/Al_2_O_3_-2.4CeO_2_-JZ, and Pd/Al_2_O_3_-xCeO_2_ catalysts by integrating peak area (summarizing in Table S2), the Pd/Al_2_O_3_-xCeO_2_ catalysts were found to have a higher concentration of surface oxygen species than the other catalysts in the following order: Pd/Al_2_O_3_-1.2CeO_2_ > Pd/Al_2_O_3_-2.4CeO_2_ > Pd/Al_2_O_3_-3.0CeO_2_. This sequence aligns with the observed catalytic activity, suggesting that the Pd-CeO_2_ interface created by the L-arginine induction method enhances the absorption of oxygen species at 200 °C and 200–400 °C. As the amount of CeO_2_ increases, the surface oxygen species obviously decrease, especially at 200–400 °C, likely because the Pd-CeO_2_ interface becomes progressively obscured by excess CeO_2_, resulting in a slight decrease in catalytic activity. Furthermore, high-temperature desorption peaks between 600 °C and 700 °C were observed in these catalysts. However, the Pd/Al_2_O_3_ sample shows a greater intensity than the other catalysts due to the decomposition of PdO_x_ species. This indicates that catalysts containing CeO_2_ have more stable PdO_x_ species, which are beneficial for oxidation activity and catalytic thermal stability [31]. Considering both the abundance of surface oxygen species and the stability of PdO_x_ species, it is anticipated that the Pd/Al_2_O_3_-xCeO_2_ catalysts will exhibit superior catalytic performance compared to other catalysts.

#### 3.1.6. Surface Composition and Metal Chemical Analysis

**Figure 3:**
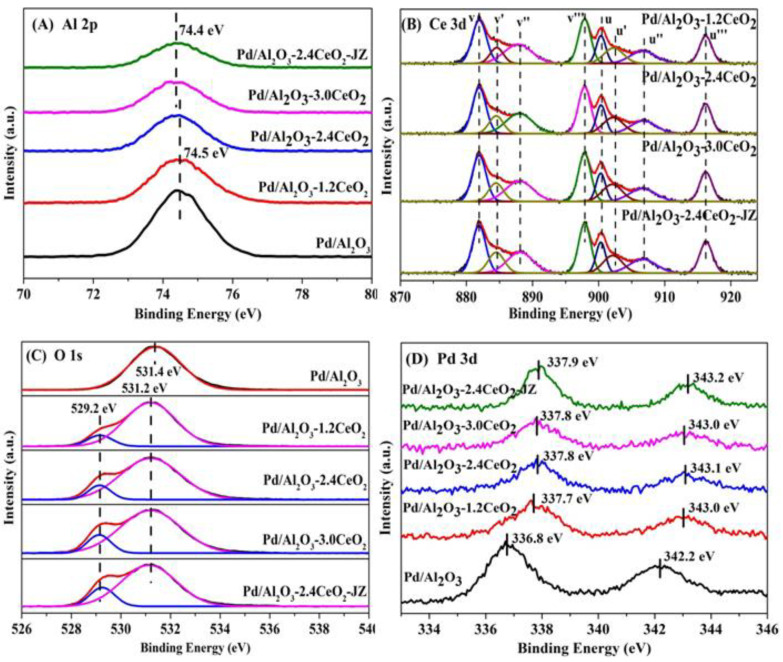
(**A**) Al 2p, (**B**) Ce 3d, (**C**) O 1s, and (**D**) Pd 3d XPS spectra of the fresh catalysts.

The surface element composition and surface species of fresh catalysts were evaluated by X-ray photoelectron spectroscopy (XPS). Figure 3 illustrates the Al 2p, O 1s, Pd 3d, and Ce 3d XPS spectra of the fresh catalysts, and the surface element compositions are summarized in Table 2. From Figure 3A, the chemical states of Al 2p spectra of all the catalysts can be described by a symmetric peak at a binding energy of 74.2–74.5 eV, which corresponds with the literature data for Al^3+^ species in Al_2_O_3_ [32,33].

Figure 3B depicts the Ce 3d XPS spectra peaks labeled u and v corresponding to the 3d_3/2_ and 3d_5/2_ spin-orbit states of the Ce cations. The signals *u’* and *v’* belong to the Ce^3+^ species, while others are ascribed to the Ce^4+^ species [34,35], indicating that both Ce^3+^ and Ce^4+^ exist in the catalysts. It could be inferred that the oxygen vacancies are formed via the reduction of Ce^4+^ to Ce^3+^ in those catalysts, which is important to their redox properties and corresponding three-way activity [36,37]. Higher concentrations of Ce^3+^ species will produce more oxygen vacancies that exist in the catalysts. In addition, the surface Ce^3+^ concentration was analyzed by integrating the peak area of their respective valence states. As listed in Table 2, the value of Ce^3+^ concentration in the sequence of Pd/Al_2_O_3_-2.4CeO_2_–JZ (19.0%) > Pd/Al_2_O_3_-3.0CeO_2_ (18.1%) > Pd/Al_2_O_3_-2.4CeO_2_ (17.7%) > Pd/Al_2_O_3_-1.2CeO_2_ (16.4%) differs from the results of catalytic activity, indicating the Ce^3+^ concentration seemingly was not the determining factor of catalytic activity.

It is widely accepted that catalysts with a higher concentration of adsorbed oxygen species are more effective for oxidation–reduction reactions [26,29]. Regarding the O 1s XPS spectra, the Pd/Al_2_O_3_ catalysts showed only the presence of absorbed oxygen. For the other catalysts, the asymmetrical O 1s spectrum could be deconvoluted into two components: the lattice oxygen (O_latt_) species at binding energies (BEs) of 529.1–529.2 eV and the surface adsorbed oxygen (O_ads_) species at BEs of 531.0–531.4 eV [38]. CeO_2_ is renowned for its excellent oxygen storage and release capabilities due to the presence of cerium ions in mixed oxidation states, which can facilitate the redox process between Ce^3+^ and Ce^4+^. Typically, O_2_ molecules can be adsorbed at the oxygen vacancies of CeO_2_ [39]. As shown in Table 2, the O_ads_/O_latt_ molar ratio for the fresh catalysts decreased in the following order: Pd/Al_2_O_3_-1.2CeO_2_ (8.1) > Pd/Al_2_O_3_-2.4CeO_2_ (7.9) > Pd/Al_2_O_3_-3.0CeO_2_ (6.0) > Pd/Al_2_O_3_-2.4CeO_2_–JZ (4.8). This order does not correspond to the variation in the Ce^3+^/Ce^4+^ molar ratio among the catalysts, suggesting that adsorbed oxygen species are not only located at the surface oxygen vacancies of CeO_2_ and the surface of Pd/Al_2_O_3_ but also that a significant amount of adsorbed oxygen exists at the interface between Pd nanoparticles and CeO_2_. This indicates that the Pd-CeO_2_ interface formed over the Pd/Al_2_O_3_-xCeO_2_ catalysts has a very strong oxygen adsorption capacity. Furthermore, the O_ads_/O_latt_ molar ratio aligns with the order of catalytic activity variation, demonstrating that the concentration of adsorbed oxygen species is the primary factor influencing catalytic activity.

Furthermore, the Pd 3d spectra of the fresh catalysts have been subjected to analysis, as shown in Figure 3D. The fresh Pd/Al_2_O_3_ catalyst exhibits a single Pd 3d_5/2_ peak at 336.8 eV, which is characteristic of PdO [40]. The Pd 3d_5/2_ spectra for Pd/Al_2_O_3_-1.2CeO_2_, Pd/Al_2_O_3_-2.4CeO_2_, Pd/Al_2_O_3_-3.0CeO_2_, and Pd/Al_2_O_3_-2.4CeO_2_–JZ are recorded at 337.7, 337.8, 337.8, and 337.9 eV, respectively. These values are shifted by 0.9, 1.0, 1.0, and 1.1eV to higher binding energies relative to PdO, suggesting a strong Pd-CeO_2_ interaction due to the electron transfer from the Pd to CeO_2_ support. This is attributed to the redox potential of Pd^2+^/Pd^0^ being lower than that of Ce^4+^/Ce^3+^ [41]. Compared to the binding energy of Pd 3d_5/2_ spectra in Pd(NO_3_)_2_ and PdCl_2_, which are 337.7 eV and 337.8 eV, respectively [42], the Pd species in the aforementioned fresh catalysts are in a highly ionic state, indicating the strong interaction between Pd species and CeO_2_. In addition, the intensity of the Pd 3d_5/2_ peak for the Pd/Al_2_O_3_-xCeO_2_ fresh catalysts containing CeO_2_ is much lower than that of the Pd/Al_2_O_3_ catalyst. This is because the Pd species are partially covered by CeO_2_ but not entirely obscured, which is consistent with the result of the metal dispersion of Pd species as presented in Table 1.

#### 3.1.7. Three-Way Catalytic Activities of Fresh Catalysts

**Figure 4:**
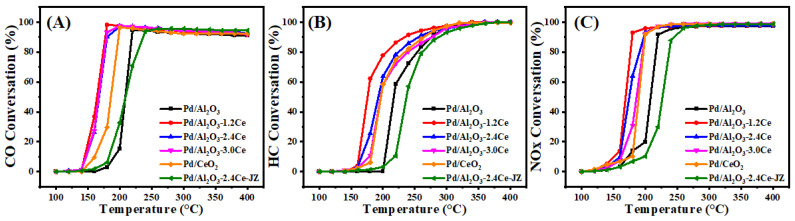
Conversions of CO (**A**), HC (**B**), and NOx (**C**) over fresh catalysts.

Figure 4 and Table S3 present the conversion results of CO, HC, and NO under stoichiometric conditions over the fresh catalysts. The *T*_90_ values, which represent the temperatures at which the 90% conversion of a given compound is achieved, are used to assess the activities of the different catalysts. Among the fresh catalysts, Pd/Al_2_O_3_-xCeO_2_ catalysts demonstrate excellent catalytic activities in the removal of three contaminations compared to the Pd/Al_2_O_3_ catalysts, with the order being Pd/Al_2_O_3_-1.2CeO_2_ ≥ Pd/Al_2_O_3_-2.4CeO_2_ ≥ Pd/Al_2_O_3_-3.0CeO_2_ > Pd/Al_2_O_3_. Notably, the Pd/Al_2_O_3_-1.2CeO_2_ catalyst displays the best catalytic activity, with a *T*_90_ of 177, 234, and 179 °C for CO, HC, and NOx, respectively. These values are lower than the *T*_90_ values of 219, 276, and 220 °C for CO, HC, and NOx over the Pd/Al_2_O_3_ catalyst, respectively. The metal–oxide interface is considered to play a prominent role in heterogeneous catalysis [43]. Analysis from the H_2_-TPR, XPS, and O_2_-TPD results indicates that the abundant Pd-CeO_2_ interfacial sites could not only absorb more oxygen species but also possessed excellent low-temperature reducibility, contributing to the three-way reaction. In addition, to elucidate the impact and advantage of the Pd-CeO_2_ interface produced by the L-arginine induction method on the activity of the Pd/Al_2_O_3_-xCeO_2_ catalysts, the catalytic performance of the Pd/CeO_2_ and Pd/Al_2_O_3_-2.4CeO_2_-JZ fresh catalysts was also investigated. The *T*_90_ values of CO and HC oxidation and NO reduction over Pd/Al_2_O_3_-2.4CeO_2_-JZ were 237, 288, and 245 °C, respectively. showing that its performance was even worse than that of Pd/Al_2_O_3_ in eliminating catalytic activity for the three pollutants. This may be due to poor selectivity, which failed to form an effective Pd-CeO_2_ interfacial active site, leading to the blockage of the active site and reduced reaction activity. In addition, the *T*_90_ values of CO and HC oxidation and NOx reduction over Pd/2.4CeO_2_-JZ were 198, 265, and 200 °C, respectively, which were also higher than the *T*_90_ of the Pd/Al_2_O_3_-xCeO_2_ catalysts. This indicates the formed Pd-CeO_2_ interface activity site is much more than that of the Pd/CeO_2_ catalyst prepared by traditional conventional synthesis methods, which have a limited-contact metal–oxide interface. In addition, the long-time stability test of the Pd/Al_2_O_3_-2.4CeO_2_ catalyst was carried out. The Pd/Al_2_O_3_-2.4CeO_2_ catalyst was evaluated by consecutively conducting the TWC test at 200 °C and 400 °C for 24 h, and it exhibited good thermal stability, as shown in Figure S11. The used Pd/Al_2_O_3_-2.4CeO_2_ catalyst after the reaction was characterized by TEM as shown in Figure S12. The morphological structure and particle size of the nanoparticles barely changed, indicating the Pd/Al_2_O_3_-2.4CeO_2_ catalyst possessed good catalytic stability.

### 3.2. Characterization of Aged Catalysts

#### 3.2.1. Morphology Analysis

**Figure 5:**
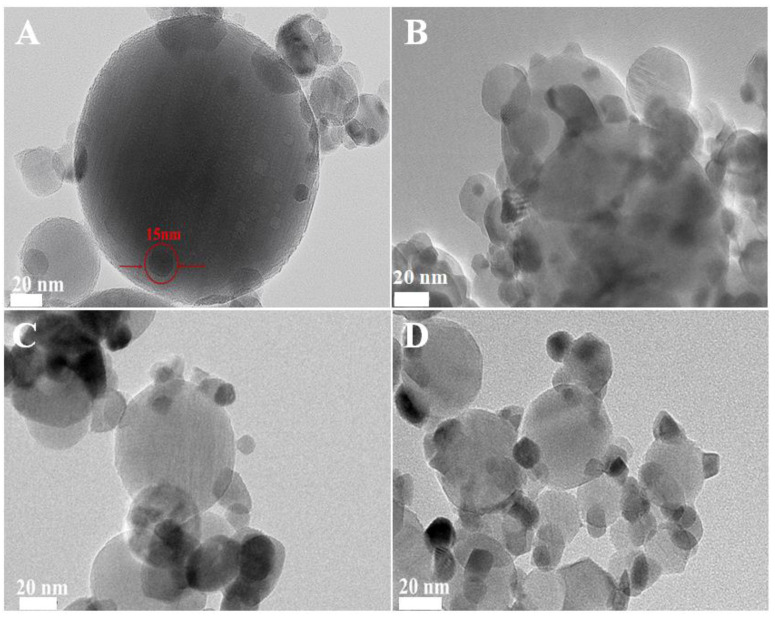
TEM of aged (**A**) Pd/Al_2_O_3_, (**B**) Pd/Al_2_O_3_-1.2CeO_2_, (**C**) Pd/Al_2_O_3_-2.4CeO_2_, (**D**) Pd/Al_2_O_3_-3.0CeO_2_ catalysts.

To further assess the thermal stability of the Pd/Al_2_O_3_-xCeO_2_ catalysts, they were subjected to calcination at 900 °C in air for 5h. The TEM and HRTEM images of the catalysts after calcination treatment are shown in Figure 5 and Figure S13. In the case of the aged Pd/Al_2_O_3_ catalyst, there was a noticeable sintering of Pd NPs, with their size increasing from 4 nm to over 10 nm, which resulted in a significant loss of catalytic activity. For the aged Pd/Al_2_O_3_-xCeO_2_ catalysts, the tiny CeO_2_ particles showed a typical agglomeration phenomenon, with their size increasing beyond 15 nm. Concurrently, lattice fringes are observed in these catalysts, where the lattice spacings of 0.32 nm and 0.26 nm correspond to the characteristic (111) or (200) planes of the fluorite phase CeO_2_, respectively. In addition, it is hard to detect the Pd NPs by TEM and HRTEM, which are present in the catalysts, as confirmed by the result of the ICP-AES (Table S1).

To clarify the distribution of Pd and CeO_2_ components after the aging treatment, EDS mapping analyses were carried out (Figure S14). However, only very few Pd species were detected, probably due to the highly dispersion of Pd species or Pd species being incorporated into the lattice of CeO_2_. It is hypothesized that Pd species were fragmented into smaller particles that redispersed in the surface of CeO_2_ due to the strong interaction between Pd species and CeO_2_ [44].

#### 3.2.2. Crystal Phase Analysis

**Figure 6:**
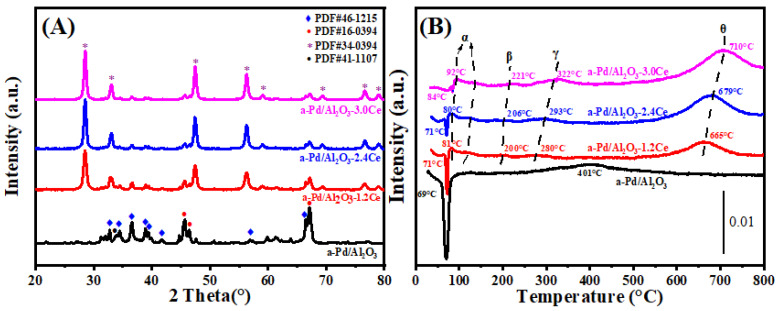
(**A**) XRD patterns and (**B**) H_2_-TPR profiles of the aged catalyst.

Following the aging treatment at 900 °C, the crystallinity of the Al_2_O_3_ remained largely unchanged, indicating that Al_2_O_3_ is relatively stable under high-temperature conditions (Figure 6). In the case of the aged Pd/Al_2_O_3_ catalyst, diffraction peaks for Pd were present at 34° (JCPDS No. 41-1107), albeit very weak, indicating that sintering had occurred in the Pd/Al_2_O_3_ catalyst. For the aged Pd/Al_2_O_3_-xCeO_2_ catalysts, no diffraction peaks of the Pd phase were observed, indicating that significant sintering of Pd did not take place, likely due to the dispersion of Pd species on the support or their incorporation into the CeO_2_ lattice. Combined with the results of the metal dispersion (Table 3), we can conclude that the Pd species were indeed highly dispersed on the support, leading to better thermal stability compared to the Pd/Al_2_O_3_ catalysts. Regarding the CeO_2_ species, the full width at a half maximum of their characteristic peaks narrowed after the aging treatment, indicating that the CeO_2_ NPs grew larger and exhibited improved crystallization, which is consistent with the results calculated using the Scherrer equation.

#### 3.2.3. Pore Structure Properties

Following the aging treatment at 900 °C, the surface area and total pore volume of Pd/Al_2_O_3_-xCeO_2_ catalysts decreased and varied from 22 to 31 m^2^/g and from 0.124 to 0.199 cm^3^/g, respectively (Table 3), due to the sintering of CeO_2_ particles. The surface area of the aged Pd/Al_2_O_3_ catalyst showed no change, indicating the Al_2_O_3_ support was stable in accordance with the results of XRD.

#### 3.2.4. Low-Temperature Reducibility Analysis

The H_2_-TPR profiles for the aged Pd/Al_2_O_3_ and Pd/Al_2_O_3_-xCeO_2_ catalysts are displayed in Figure 6B. A negative peak in the range of 69–85 °C is observed for the aged catalysts, with the aged Pd/Al_2_O_3_ sample exhibiting the most significant peak. This peak is generally attributed to the decomposition of palladium hydride, indicating the presence of larger Pd crystallites with an enhanced ability to adsorb H_2_ via hydride formation [45]. Larger Pd particles exhibit more obvious decomposition peaks of palladium hydride, as hydride formation is a bulk phenomenon. The H_2_ release for the negative peak follows the order: Pd/Al_2_O_3_-900 (80.9 μmol/g_cat_) > Pd/Al_2_O_3_-500 (26 μmol/g_cat_) > Pd/Al_2_O_3_-1.2CeO_2_ (14.5 μmol/g_cat_) > Pd/Al_2_O_3_-2.4CeO_2_ (4 μmol/g_cat_) > Pd/Al_2_O_3_-3.0CeO_2_ (0.84 μmol/g_cat_). This indicates that the Pd species in Pd/Al_2_O_3_-xCeO_2_ catalysts did not undergo severe sintering and maintained a higher dispersion, which is consistent with the results detected by the CO pulse method listed in Table 3. In comparison to the fresh Pd/Al_2_O_3_ sample, an additional reduction peak emerges at 401 °C, with an H_2_ consumption of 95.8 μmol/gcat for the aged Pd/Al_2_O_3_ sample. This suggests that more PdO species were formed, which are difficult to reduce at low temperatures after calcination at 900 °C.

It is noteworthy that after the aging treatment, the intensity of the low-temperature reduction peaks (α) for the Pd/Al_2_O_3_-xCeO_2_ catalysts was obviously reduced, and the peaks shifted to higher temperatures. This is likely due to the severe sintering of CeO_2_, which may have disrupted the Pd-CeO_2_ interface formed by the L-arginine induction method. However, aside from the Pd/Al_2_O_3_-1.2CeO_2_ sample, the catalytic performance of the Pd/Al_2_O_3_-xCeO_2_ catalysts did not change markedly after aging at 900 °C. The lower H_2_ consumption of the negative peak and the higher dispersion of Pd species, as detected by the CO pulse method, suggest that no obvious sintering occurred for the Pd species. The reduction peaks (β) correspond to the reduction of PdO_x_ species that are embedded within the CeO_2_ lattice in the form of a -Pd^2+^-O^2−^-Ce^4+^ linkage, which occurs during the aging process [46]. The reduction peaks (γ) shifted to lower temperatures, and the H_2_ consumption decreased, possibly due to the high dispersion of Pd on CeO_2_ and a reduction in the Pd-CeO_2_ interface. In addition, the intensity of the reduction of peak (θ) increased, and peaks shifted to higher temperatures, which could be influenced by the dispersion state and sintering of CeO_2_. Additionally, the bulk reduction peaks of CeO_2_ beyond 800 °C also shift to higher temperatures as a result of the severe sintering of CeO_2_.

Due to the interaction between Pd species and CeO_2_, leading to the Pd species being held in a high dispersion state, generally the higher Pd dispersion and smaller Pd particle size produce a higher active surface, resulting in a superior catalytic activity [29].

#### 3.2.5. Surface Composition and Metal Chemical Analysis

**Figure 7:**
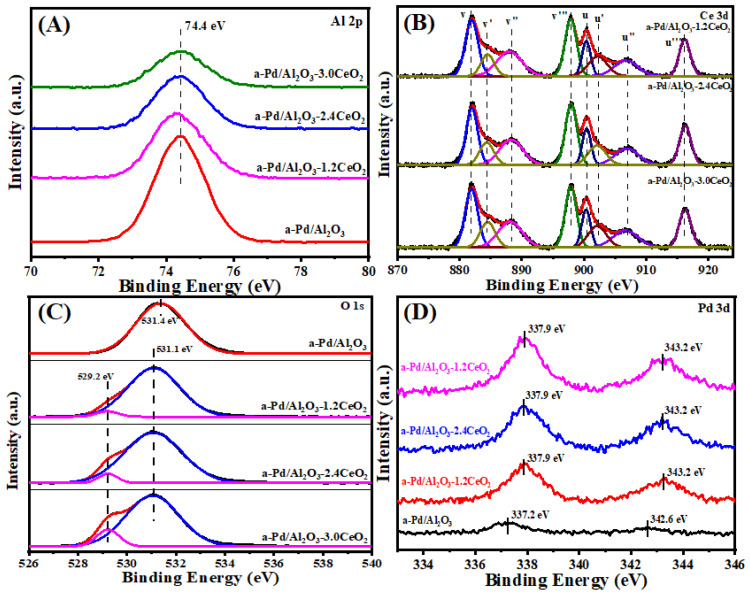
(**A**) Al 2p, (**B**) Ce 3d, (**C**) O 1s, and (**D**) Pd 3d XPS spectra of the aged catalysts.

Figure 7 illustrates the Al 2p, O 1s, Pd 3d, and Ce 3d XPS spectra of the fresh catalysts, with the surface element composition summarized in Table 4. The chemical states of Al 2p spectra remain almost unchanged before and after catalyst aging. Regarding the Ce 3d XPS spectra, both valence states of Ce^3+^ and Ce^4+^ continue to coexist in the catalyst, with no obvious decrease in the Ce^3+^ concentration observed. This could be attributed to the incorporation of Pd NPs into the lattice of CeO_2_ particles, leading to the generation of oxygen defects. Meanwhile, the O_ads_/O_latt_ ratio increases compared to that of the fresh catalysts, indicating a decrease in lattice oxygen concentration and the creation of more oxygen defects [47]. Furthermore, after calcination at 900 °C in air, the Pd 3d value over the Pd/Al_2_O_3_ catalyst shifts from 336.8 to 337.2 eV, indicating the formation of a higher oxidation state of Pd and the occurrence of the sintering phenomenon among Pd species during the aging process [48], which is in agreement with the result of the TEM images. For the Pd/Al_2_O_3_-xCeO_2_ catalysts, there is a significant change in the peak position of Pd 3d, even if Pd enters the lattice of CeO_2_ due to the similar binding energy of Ce_1-x_Pd_x_O_2-δ_ solid solution around 337.9 eV [49,50,51]. At this point, the intensity of Pd/Al_2_O_3_-xCeO_2_ catalysts increases, indicating the Pd species are highly dispersed on the surface of CeO_2_. This further confirms the strong interaction between Pd and CeO_2_.

#### 3.2.6. Three-Way Catalytic Activities of Aged Catalysts

**Figure 8:**
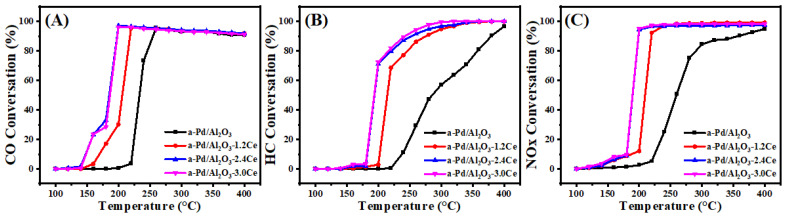
Conversion of CO (**A**), HC (**B**), and NOx (**C**) over the aged catalysts.

Figure 8 and Table S4 present the conversion results of CO, HC, and NOx under stoichiometric conditions over aged catalysts. After calcination at 900 °C, the *T*_90_ values for CO and HC oxidation and NOx reduction over the aged Pd/Al_2_O_3_ catalysts increased by ~36, 104, and 139 °C, respectively, compared to the fresh samples. For the aged Pd/Al_2_O_3_-1.2CeO_2_ catalysts, the *T*_90_ value for CO, HC, and NO increased by 41, 42, and 40 °C, respectively. The large change in the activity of the Pd/Al_2_O_3_-1.2CeO_2_ catalyst may be due to the destruction of the Pd-CeO_2_ interface and some sintering of the Pd nanoparticles. However, the catalytic activities of the aged Pd/Al_2_O_3_-2.4CeO_2_ and Pd/Al_2_O_3_-3.0CeO_2_ catalysts remained unchanged, except for an increase of about 20 °C in the *T*_90_ values for CO, indicating their outstanding thermal stability. Surprisingly, the *T*_90_ values for HC in the Pd/Al_2_O_3_-3.0CeO_2_ catalyst decreased by about 35 °C. Combining the catalytic activity results with TEM images of the Pd/Al_2_O_3_-xCeO_2_ catalysts, it was observed that Pd NPs could not be observed by HRTEM in those catalysts, which can be attributed to their high dispersion, consistent with the metal dispersion results. In addition, increasing the CeO_2_ content enhances metal dispersion upon calcination at high temperatures. At the same time, some Pd species are incorporated into the CeO_2_ lattice in the Pd/Al_2_O_3_-xCeO_2_ catalysts, as evidenced by the Ce 3d and O 1s XPS spectra. In conclusion, the high dispersion of Pd species and the presence of a large number of oxygen vacancies are the key factors contributing to the better catalytic activities of the aged Pd/Al_2_O_3_-xCeO_2_ catalysts after high-temperature treatment.

## 4. Conclusions

We have successfully demonstrated a novel approach to precisely apply a catalyst, where CeO_2_ selectively decorates Pd nanoparticles supported on Al_2_O_3_ using the L-arginine induction method. This technique significantly enhances the catalytic activity and redox properties of the Pd/Al_2_O_3_-xCeO_2_ catalyst by forming and expanding the contact interface between Pd and CeO_2_. EDS mapping confirmed that Pd nanoparticles were indeed selectively decorated by CeO_2_, creating the Pd-CeO_2_ interface. O_2_-TPD and XPS results revealed that a larger amount of absorbed oxygen was present at the Pd-CeO_2_ interface. After calcination at 900 °C for 5 h, the Pd/Al_2_O_3_ catalyst underwent severe sintering, leading to a deterioration in redox properties. In contrast, the aged Pd/Al_2_O_3_-xCeO_2_ catalyst showed, according to TEM, XRD, and BET results, that the CeO_2_ severely sintered at high temperatures. However, Pd species were not detectable by XRD and TEM, indicating that there was no sintering phenomenon for the Pd species. XPS and metal dispersion studies revealed that Pd species were highly dispersed on the CeO_2_ support, with some Pd species incorporated into the CeO_2_ lattice, resulting in no significant change in the number of oxygen vacancies. Thus, it is evident that the metal–oxide interface and the metal–support interaction plays crucial roles in improving catalyst activity and promoting the redispersion of Pd species at high temperatures. This approach is an effective way to regulate catalytic activity and enhance the thermal stability of catalysts at elevated temperatures.

## Figures and Tables

**Table 1:** BET surface areas, pore volumes, average sizes (D_CeO2_), and dispersion of the as-obtained catalysts.

Fresh Catalysts	Surface Area (m^2^·g^−1^)	Pore Volume (cm^3^·g^−1^)	D_CeO2_^a^ (nm)	Dispersion of Pd ^b^ (%)
Pd/Al_2_O_3_	34	0.193	—	27.8
Pd/Al_2_O_3_-1.2CeO_2_	56	0.248	5.5	18.7
Pd/Al_2_O_3_-2.4CeO_2_	62	0.231	6.7	16.4
Pd/Al_2_O_3_-3.0CeO_2_	71	0.220	7.1	15.2
Pd/Al_2_O_3_-2.4CeO_2_-JZ	46	0.140	7.5	22.1

^a^ Data were obtained according to the Scherrer equation using the FWHM of CeO_2_ in the XRD patterns. ^b^ Data were obtained by the CO pulse method.

**Table 2:** H_2_ consumption and surface element composition of the fresh catalysts.

Fresh Catalysts	Reducibility of Fresh Catalysts (H_2_ Consumption (μmol/gcat))					Fresh-Surface Element Composition Molar Ratio (%)	
	Negative Peak	<200 °C	200–250 °C	250–350 °C	500–750 °C	O_ads_/O_latt_	Ce^3+^/(Ce^3+^ + Ce^4+^)
Pd/Al_2_O_3_	−26.0	—	6.5	—	—	—	—
Pd/Al_2_O_3_-1.2CeO_2_	—	88.7	—	18.2	43.2	8.1	16.4
Pd/Al_2_O_3_-2.4CeO_2_	—	76.8	—	21.8	102.1	7.9	17.7
Pd/Al_2_O_3_-3.0CeO_2_	—	69.9	—	41.5	112.2	6.0	18.1
Pd/Al_2_O_3_-2.4CeO_2_-JZ	—	225.7	—	29.5	78.2	4.8	19.0

**Table 3:** BET surface areas, pore volumes, average sizes (D_CeO2_), and dispersion of the aged catalysts.

Aged Catalysts	Surface Area (m^2^·g^−1^)	Pore Volume (cm^3^·g^−1^)	D_CeO2_^a^ (nm)	Dispersion of Pd ^b^ (%)
Pd/Al_2_O_3_	34	0.215	—	13.0
Pd/Al_2_O_3_-1.2CeO_2_	30	0.199	19.2	44.7
Pd/Al_2_O_3_-2.4CeO_2_	26	0.141	24.6	53.3
Pd/Al_2_O_3_-3.0CeO	31	0.136	25.8	58.4

^a^ Data were obtained according to the Scherrer equation using the FWHM of CeO_2_ in the XRD patterns. ^b^ Data were obtained by the CO pulse method.

**Table 4:** H_2_ consumption and surface element composition of the aged catalysts.

Aged Catalysts	Reducibility of Fresh Catalysts (H_2_ Consumption (μmol/gcat))					Aged-Surface Element Composition Molar Ratio (%)	
	Negative Peak	<200 °C	200–250 °C	250–350 °C	500–750 °C	O_ads_/O_latt_	Ce^3+^/(Ce^3+^ + Ce^4+^)
a-Pd/Al_2_O_3_	−80.9	—	—	95.8	—	—	—
a-Pd/Al_2_O_3_-1.2CeO_2_	−14.5	11.9	2.6	9.0	112.9	21.8	15.9
a-Pd/Al_2_O_3_-2.4CeO_2_	−4.0	18.8	3.5	16.2	169.1	16.7	17.6
a-Pd/Al_2_O_3_-3.0CeO_2_	−0.84	24.9	3.9	29.7	185.4	7.2	18.1

## Data Availability

Data are contained within this article.

## Supplementary Materials

The following supporting information can be downloaded at: https://www.mdpi.com/article/10.3390/nano15030197/s1, Figure S1: HRTEM image of irregular Pd nanoparticles; Figure S2: EDS mapping images of fresh 4 nm Pd/Al_2_O_3_-1.2CeO_2_ catalyst; Figure S3: TEM images of 12 nm (A) and 19 nm (B) Pd nanoparticles; Figure S4: EDS mapping images of 12 nm Pd/Al_2_O_3_-1.2CeO_2_ fresh catalyst; Figure S5: Local EDS mapping images of 12 nm Pd/Al_2_O_3_-1.2CeO_2_ fresh catalyst; Figure S6: EDS mapping images of 19 nm Pd/Al_2_O_3_-1.2CeO_2_ fresh catalyst; Figure S7: TEM images of Pd/Al_2_O_3_-2.4CeO_2_-JZ fresh catalyst; Figure S8: EDS mapping images of 19 nm Pd/Al_2_O_3_-2.4CeO_2_-JZ fresh catalyst; Figure S9: EDS mapping images of 19 nm Pd/Al_2_O_3_-1.2CeO_2_-JZ fresh catalyst; Figure S10: Adsorption–desorption isotherms of fresh catalysts; Figure S11: Long-time stability tests by Pd/Al_2_O_3_-2.4CeO_2_ catalyst at 200 °C (A) and 400 °C (B); Figure S12: TEM images of used Pd/Al_2_O_3_-2.4CeO_2_ catalyst after long-time stability tests at 400 °C for 24 h; Figure S13: HRTEM images of aged (A) Pd/Al_2_O_3_-1.2CeO_2_, (B) Pd/Al_2_O_3_-2.4CeO_2_, and (C) Pd/Al_2_O_3_-3.0CeO_2_catalysts; Figure S14: EDS mapping images of aged Pd/Al_2_O_3_-1.2CeO_2_ catalysts; Table S1: Real loading of Pd determined by the ICP-AES technique; Table S2: Oxygen desorption of the fresh catalysts; Table S3: The temperature required to reach 90% conversion of CO, HC, and NO over fresh catalysts; Table S4: The temperature required to reach 90% conversion of CO, HC, and NO over aged catalysts.
