# Supplementary material for: Tailoring Metal–Oxide Interfaces via Selectively CeO2-Decorated Pd Nanocatalysts with Enhanced Catalytic Performance
Source: Nanomaterials (Basel). 2025 Jan 27;15(3):197. doi: 10.3390/nano15030197 (PMC11820916; doi:10.3390/nano15030197)
Supplement: Supplementary file 1 [file nanomaterials-15-00197-s001.zip › nanomaterials-3430303-supplementary.pdf]

nanomaterials

MDPI

Supplementary Materials

Tailoring Metal–Oxide Interfaces via Selectively CeO_2-Decorated Pd Nanocatalysts with Enhanced Catalytic Performance

Ziwen Liu [1,2], Guizhen Zhang [1,*], Lijuan Niu [3], Zaicheng Sun [4] and Zhenguo Li [5,*] and Hong He [1]

[1] Beijing Key Laboratory for Green Catalysis and Separation, College of Materials Science & Engineering, Beijing University of Technology, Beijing 100124, China; liuzw2023.chji@sinopec.com (Z.L.)
[2] Institute of Engineering Technology, Sinopec Catalyst Co., Ltd., Beijing 101111, China
[3] Carbon Energy Technology Co., Ltd., Beijing 102400, China; nlj@carbonenergy.com.cn
[4] Center of Excellence for Environmental Safety and Biological Effects, Beijing Key Laboratory for Green Catalysis and Separation, Department of Chemistry, College of Chemistry and Life Science, Beijing University of Technology, Beijing 100124, China; sunzc@bjut.edu.cn
[5] China Automotive Technology and Research Center Co., Ltd., Tianjin 300300, China
* Correspondence: zhangguizhen@bjut.edu.cn (G.Z.); lizhenguo@catarc.ac.cn (Z.L.)

Catalyst characterizations

Transmission electron microscopy (TEM) images of the samples were recorded on a JEOL-2100F electron microscope operated at 200 kV. The elemental compositions of the samples were characterized via an energy-dispersive X-ray spectroscopy (EDS) detector and the FEI Titan G2 60–300 (microscopy) equipped with a Cs-corrected probe featuring a large solid angle (~1 sr) that enables X-ray count rates over 100 kcps.

The specific surface areas and pore volumes were performed at -196 °C on an ASAP 2460 after the sample was evacuated at 250 °C for 3 h. Brunauer-Emmett-Teller (BET) and Barret-Joyner-Halenda (BJH) equations were used for the determination of surface area and pore volumes, respectively.

Powder X-ray diffraction (XRD) patterns of the catalysts were collected on a Bruker/AXS (D8 ADVANCE) diffractometer with Cu Kα radiation (λ = 1.5418 Å) operated at 40 kV and 35 mA.

The elemental analysis was obtained with a Varian Vista MPX inductively coupled plasma atomic emission spectrometer (ICP-AES).

The X-ray photoelectron spectroscopy (XPS) spectrums of the materials were recorded in a Thermo Fisher ESCALAB 250 xi photoelectron spectroscope, using Al Kα radiation (1486.6 eV). Binding energies were measured with a precision of ±0.5 eV and calibrated to C (1s) at 284.8 eV.

H_2-TPR was performed on a chemisorption analyzer (Micromeritics, AutoChem II 2920). 50 mg of the catalyst was pretreated in 20% O_2-N_2 at 300 °C for 1 h and then cooled to room temperature. Before measurement, the sample was purged by He for 30 min. When the baseline remained unchanged, the TPR profile of the catalyst was obtained by heating the sample from ambient temperature to 900 °C in a flow of 10% H_2/He (30 mL/min) with a temperature ramp of 10 °C/min. The reducing gas was cooled in a bath of a mixture of isopropanol and liquid nitrogen to condense the water generated from the reduction of the sample.

The metal dispersion of Pd (D_{Pd}) was measured by using a modified pulsed CO technique, which was carried out on a chemisorption analyzer (Micromeritics, AutoChem II 2920). Typically, 50 mg of catalyst was used for the test. The sample was first pretreated in 20% O_2-N_2 at 300 °C for 30 minutes and then cooled to room temperature. Next, the sample was reduced in a 30 mL/min flow of 10% H_2/Ar from room temperature to 200 °C. Waiting for cooling down to 50 °C, the sample was exposed to gases in the following sequence of steps: (i) He (5 min); (ii) 20% O_2-N_2 (5 min); (iii) CO_2 (10 min); (iv) He (20 min); (v) 10% H_2/He (5 min). Finally, 10% CO/He was pulsed every 2 min until the sample adsorption was saturated.

Temperature-programmed desorption of oxygen (O_2-TPD) was performed by mass spectrometry (MS, Hidden QGA). Temperature-programmed desorption of oxygen (O_2-TPD) measurements were conducted on a conventional

flow apparatus. The sample was pretreated in 20% O_2/N_2 (30 ml/min) at 250 °C for 1 h and then cooled to ambient temperature. A flow of Ar (purity 99.999%) was then introduced to purge away the adsorbed oxygen until the stabilization of MS baseline, and the oxygen desorbed was carried on from ambient temperature to 900 °C at a rate of 10 °C/min.

Table S1. Real loading of Pd determined by the ICP-AES technique

Catalysts	Pd loading (wt%)	
	Fresh catalysts	Aged catalysts
Pd/Al_2O_3	0.93	0.85
Pd/Al_2O_3-1.2CeO_2	0.80	0.76
Pd/Al_2O_3-2.4CeO_2	0.84	0.80
Pd/Al_2O_3-3.0CeO_2	0.79	0.78
Pd/CeO_2	0.86	—
Pd/Al_2O_3-2.4CeO_2-JZ	0.89	—

Table S2. Oxygen desorption of the fresh catalysts

Fresh catalysts	Area of O_2 desorption (× 10^{-8})			Area Ratio (%)	
	< 200 °C	200-400 °C	Total	< 200 °C	200-400 °C
Pd/Al_2O_3	0.59	0.21	0.80	73.7	26.3
Pd/Al_2O_3-1.2CeO_2	1.04	0.54	1.58	65.7	34.3
Pd/Al_2O_3-2.4CeO_2	1.10	0.36	1.46	75.4	24.6
Pd/Al_2O_3-3.0CeO_2	0.94	0.13	1.28	73.4	10.0
Pd/Al_2O_3-2.4CeO_2-JZ	0.80	0.20	1.00	79.6	20.4

Table S3. The temperature required to reach 90% conversion of CO, HC, and NO over fresh catalysts

Fresh catalysts	Fresh (T_{90}/°C)		
	CO	HC	NO
Pd/Al_2O_3	219	276	220
Pd/Al_2O_3-1.2CeO_2	177	234	179
Pd/Al_2O_3-2.4CeO_2	180	256	198
Pd/Al_2O_3-3.0CeO_2	179	277	199
Pd/CeO_2	198	265	200
Pd/Al_2O_3-2.4CeO_2-JZ	237	288	245

Table S4. The temperature required to reach 90% conversion of CO, HC, and NO over aged catalysts

aged catalysts	Aged (T_{90}/°C)		
	CO	HC	NO
Pd/Al_2O_3	255	380	358
Pd/Al_2O_3-1.2CeO_2	218	276	219
Pd/Al_2O_3-2.4CeO_2	198	253	199
Pd/Al_2O_3-3.0CeO_2	198	242	199

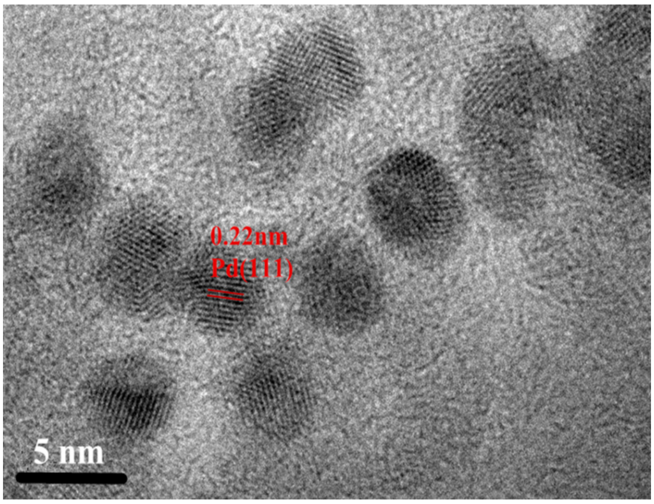

Figure. S1. HRTEM image of irregular Pd nanoparticles.

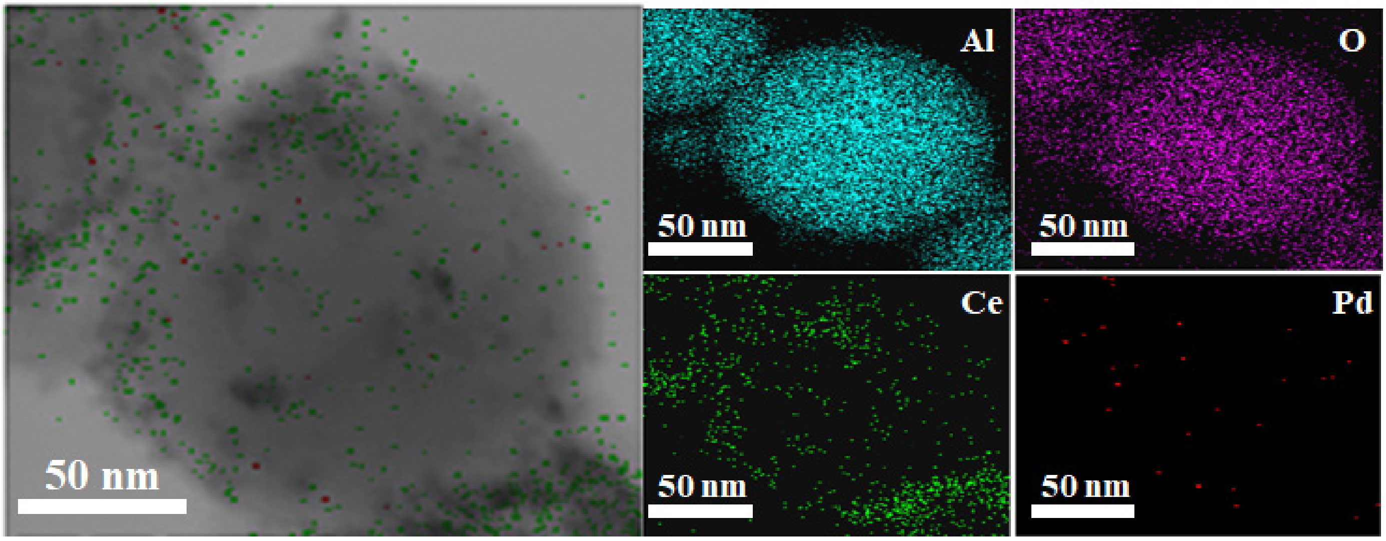

Figure. S2. EDS-mapping images of fresh 4 nm Pd/Al_2O_3-1.2CeO_2 catalyst.

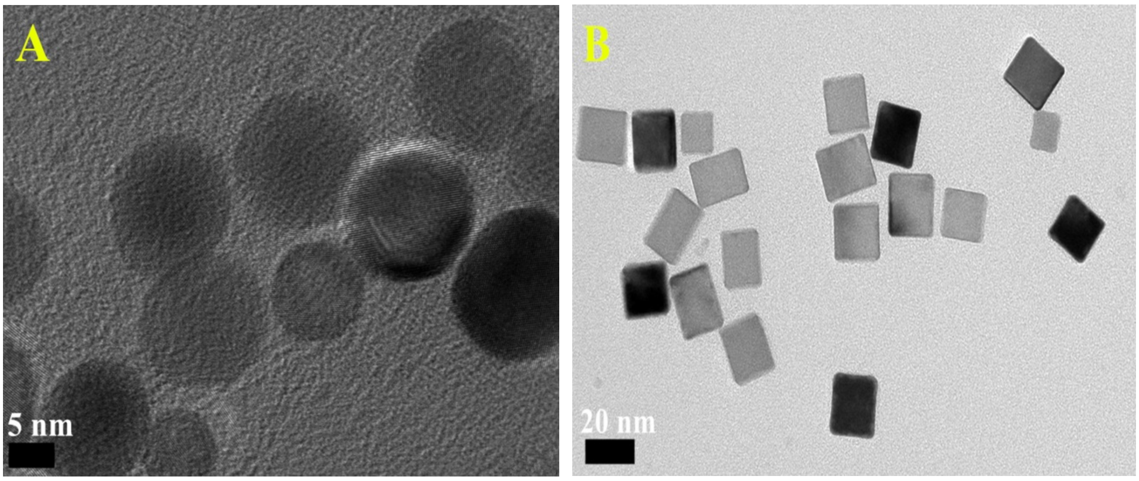

Figure. S3. TEM images of 12 nm (A) and 19 nm (B) Pd nanoparticles.

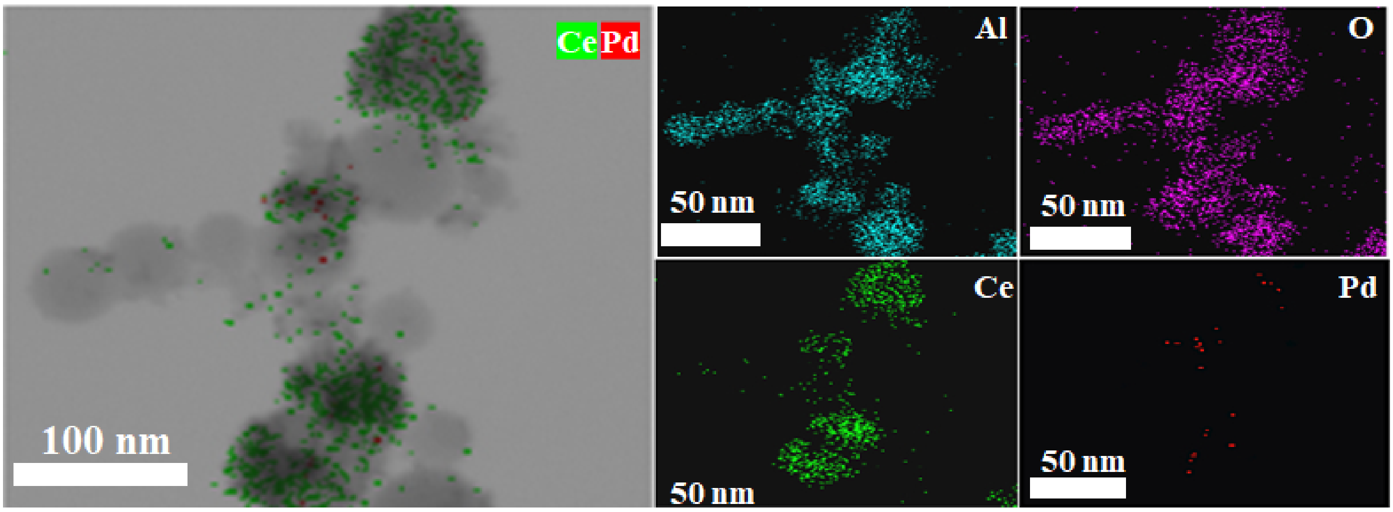

Figure. S4. EDS-mapping images of 12 nm Pd/Al_2O_3-1.2CeO_2 fresh catalyst

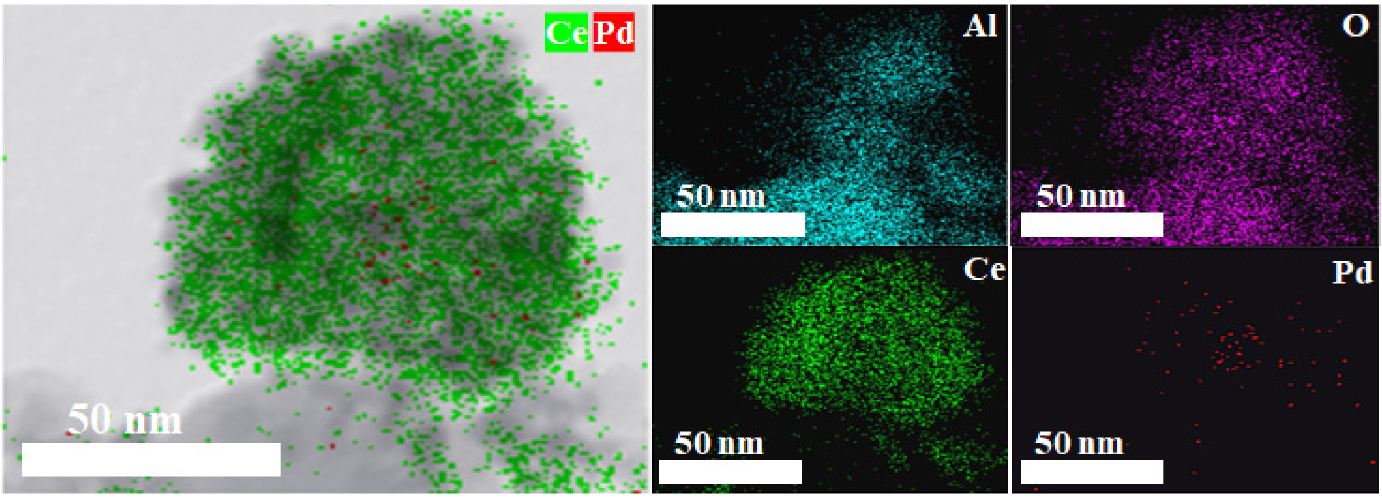

Figure. S5. Local EDS-mapping images of 12 nm Pd/Al_2O_3-1.2CeO_2 fresh catalyst.

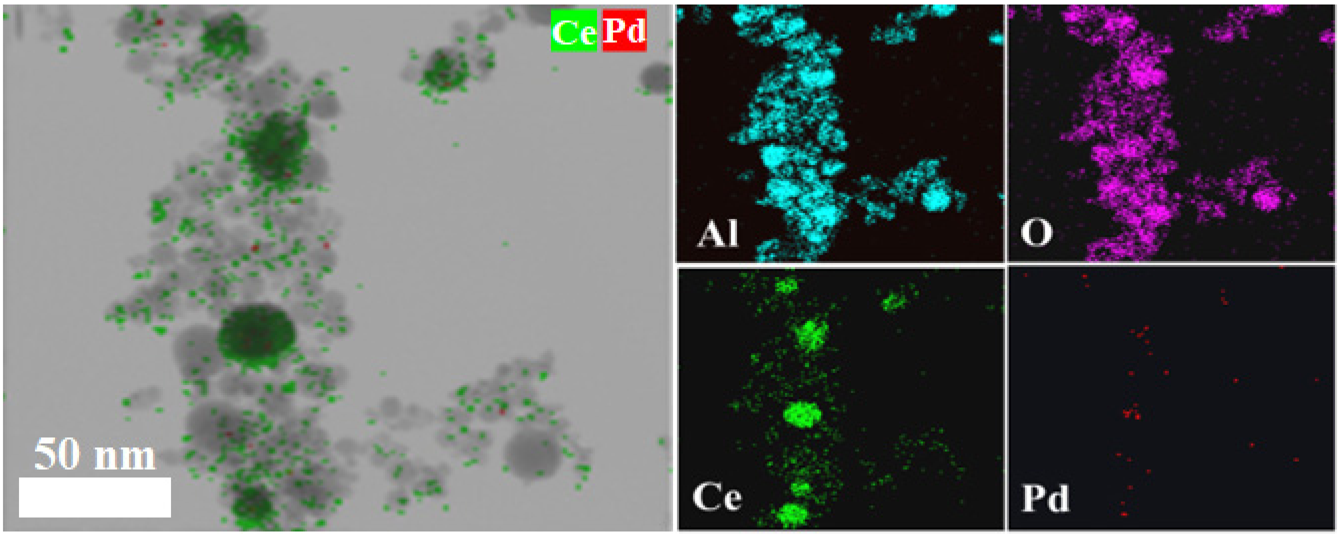

Figure. S6. EDS-mapping images of 19 nm Pd/Al_2O_3-1.2CeO_2 fresh catalyst.

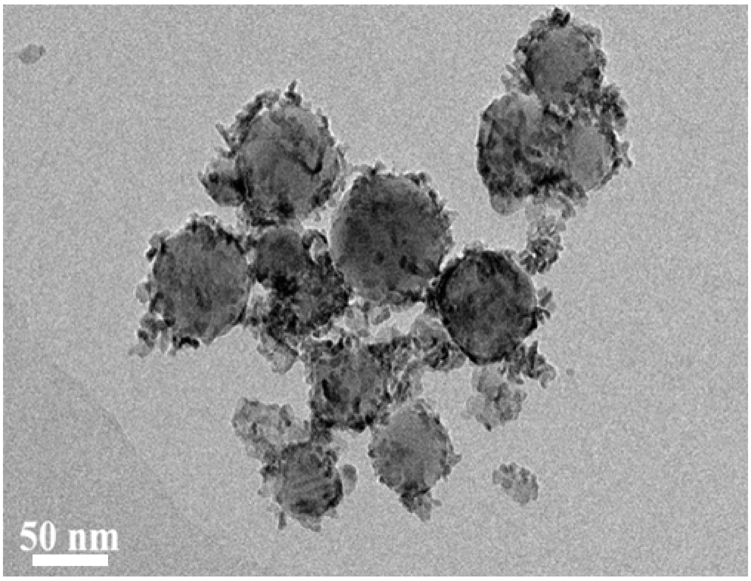

Figure. S7. TEM images of Pd/Al_2O_3-2.4CeO_2-JZ fresh catalyst.

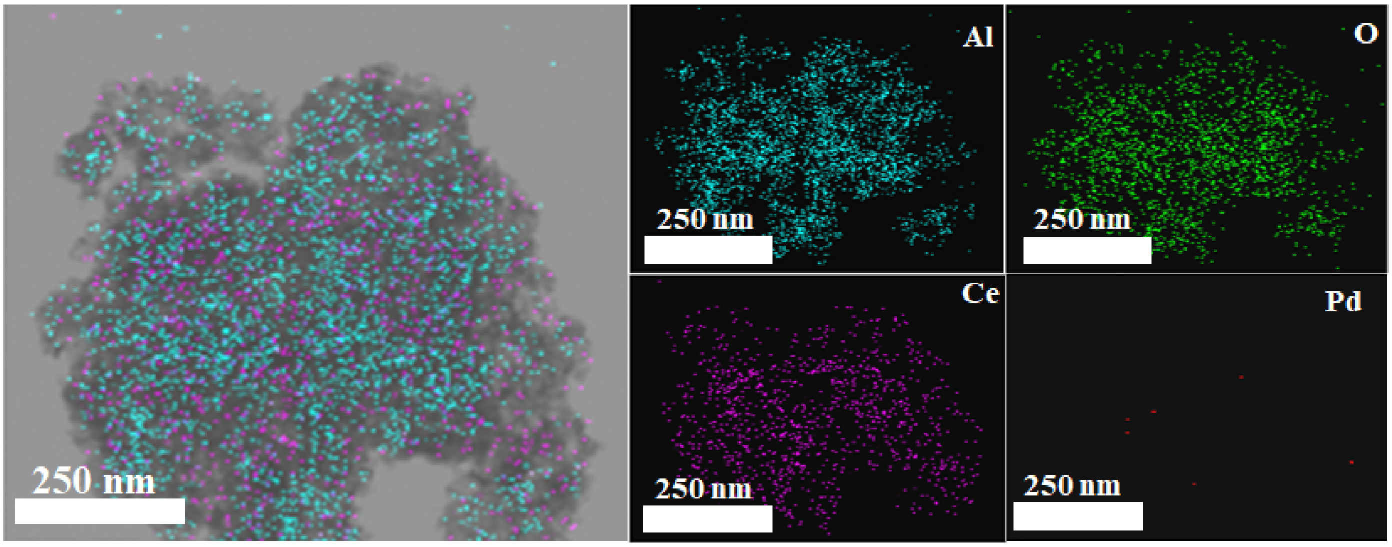

Figure. S8. EDS-mapping images of 19 nm Pd/Al_2O_3-2.4CeO_2-JZ fresh catalyst.

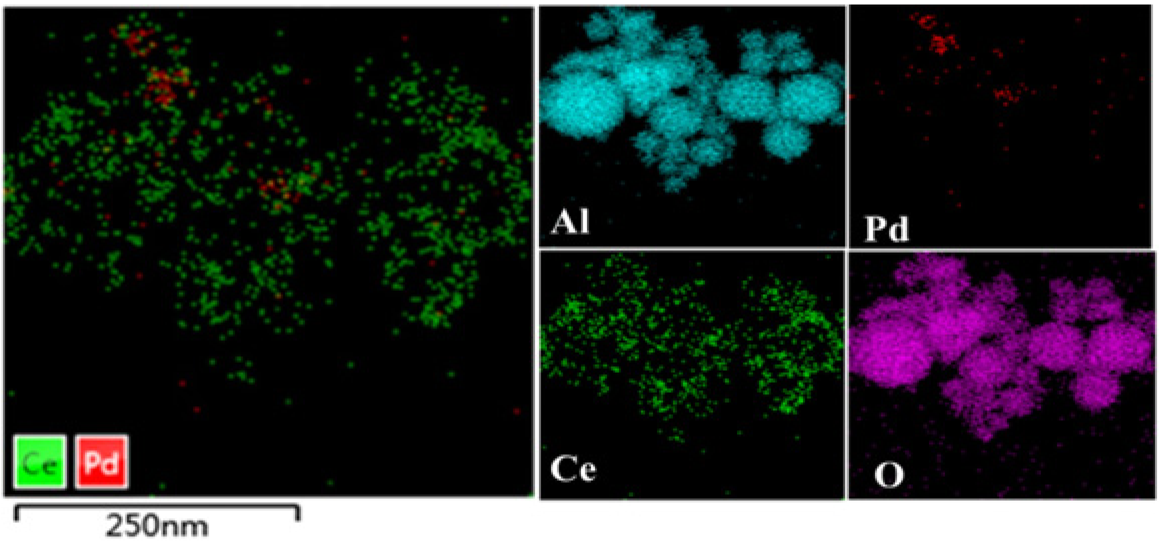

Figure. S9. EDS-mapping images of 19 nm Pd/Al_2O_3-1.2CeO_2-JZ fresh catalyst.

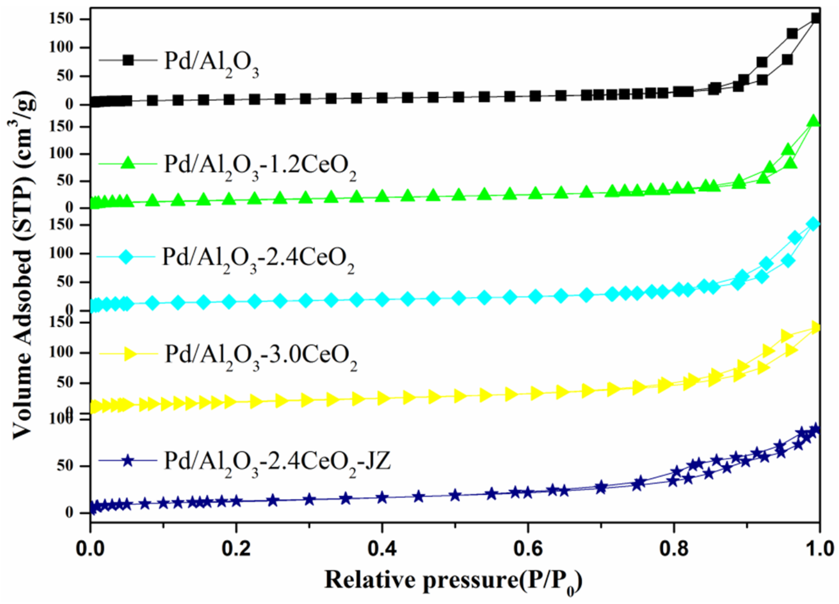

Figure. S10. Adsorption-desorption isotherms of fresh catalysts

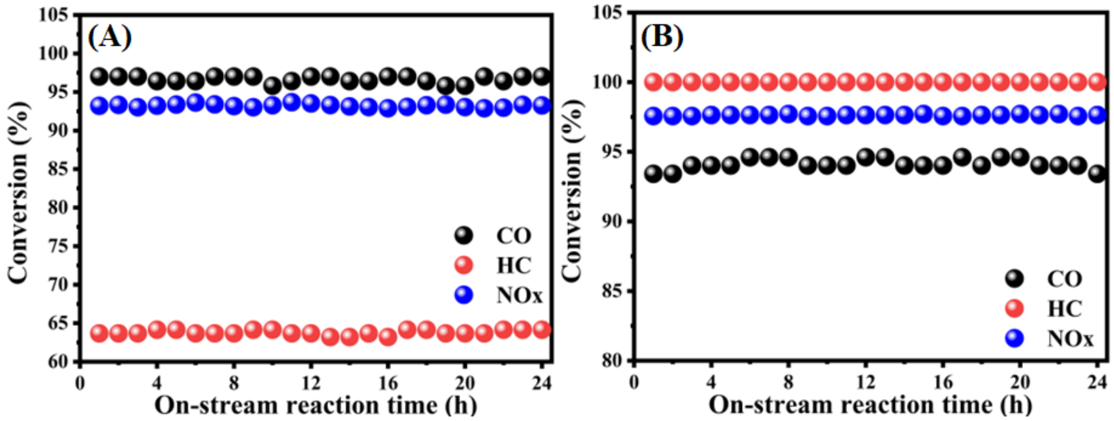

Figure. S11. Long-time stability tests by Pd/Al_2O_3-2.4CeO_2 catalyst at 200 °C (A) and 400 °C (B).

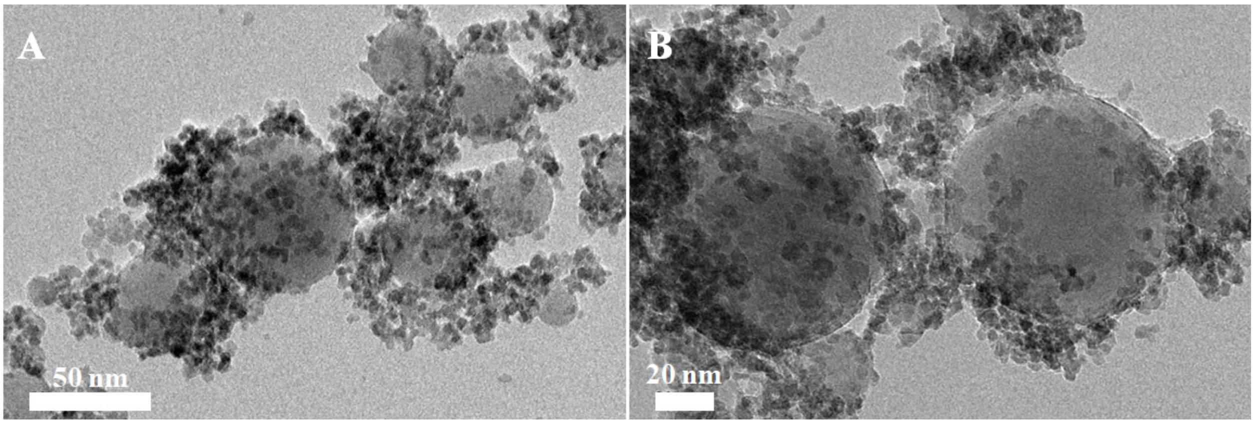

Figure. S12. TEM images of used Pd/Al_2O_3-2.4CeO_2 catalyst after long-time stability tests at 400 °C for 24 h.

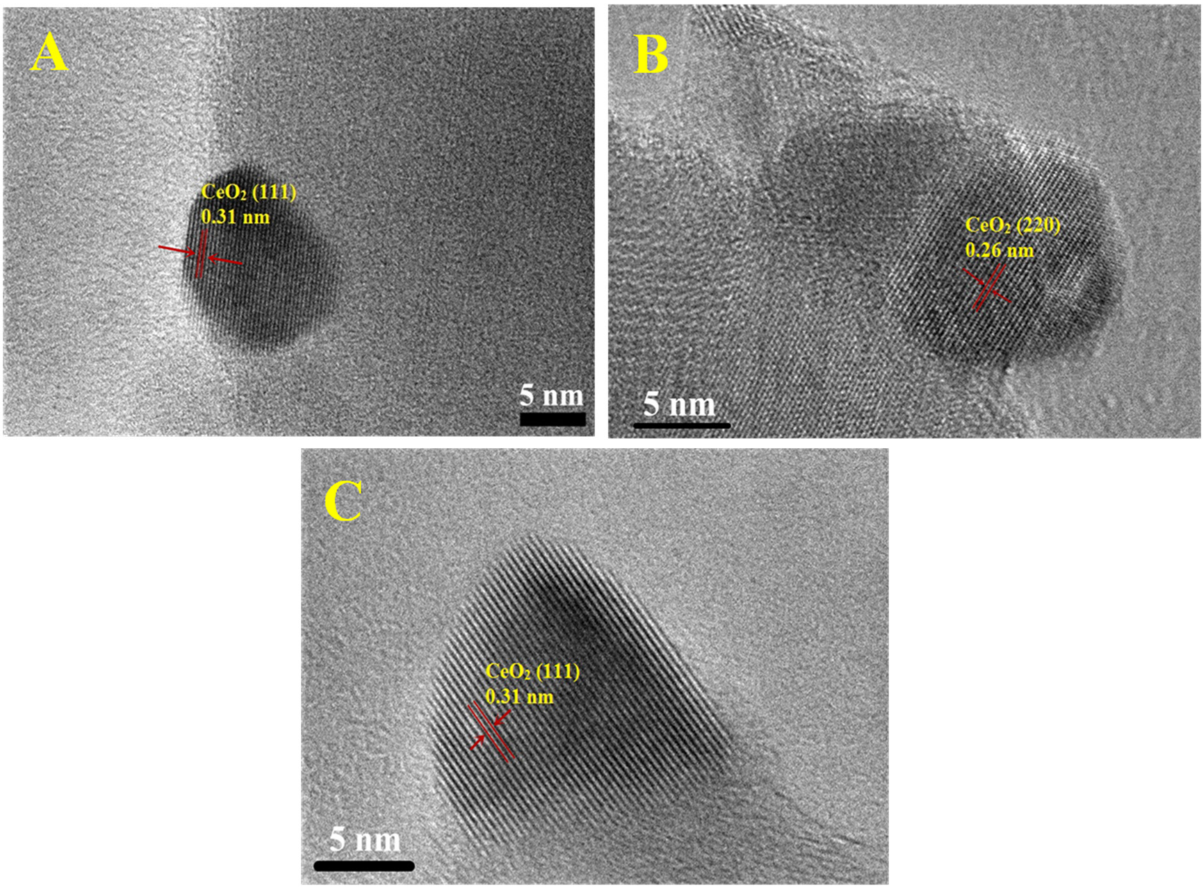

Figure. S13. HRTEM images of aged (A) Pd/Al_2O_3-1.2CeO_2, (B) Pd/Al_2O_3-2.4CeO_2, and (C) Pd/Al_2O_3-3.0CeO_2 catalysts.

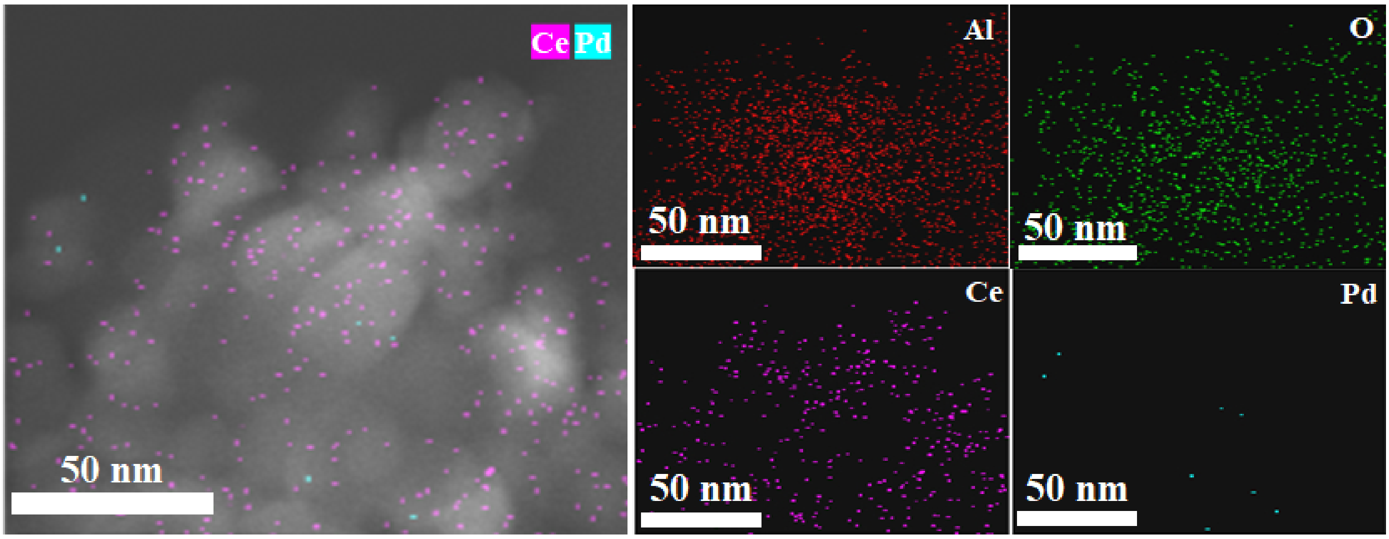

Figure. S14. EDS-mapping images of aged Pd/Al_2O_3-1.2CeO_2 catalysts
